# Reactive Nitrogen Species in Mitochondria and Their Implications in Plant Energy Status and Hypoxic Stress Tolerance

**DOI:** 10.3389/fpls.2016.00369

**Published:** 2016-03-24

**Authors:** Kapuganti Jagadis Gupta, Abir U. Igamberdiev

**Affiliations:** ^1^National Institute of Plant Genome ResearchNew Delhi, India; ^2^Department of Biology, Memorial University of Newfoundland, St. John’sNL, Canada

**Keywords:** peroxynitrite, nitric oxide, superoxide, hypoxia, mitochondria

## Abstract

Hypoxic and anoxic conditions result in the energy crisis that leads to cell damage. Since mitochondria are the primary organelles for energy production, the support of these organelles in a functional state is an important task during oxygen deprivation. Plant mitochondria adapted the strategy to survive under hypoxia by keeping electron transport operative even without oxygen via the use of nitrite as a terminal electrons acceptor. The process of nitrite reduction to nitric oxide (NO) in the mitochondrial electron transport chain recycles NADH and leads to a limited rate of ATP production. The produced ATP alongside with the ATP generated by fermentation supports the processes of transcription and translation required for hypoxic survival and recovery of plants. Non-symbiotic hemoglobins (called phytoglobins in plants) scavenge NO and thus contribute to regeneration of NAD^+^ and nitrate required for the operation of anaerobic energy metabolism. This overall operation represents an important strategy of biochemical adaptation that results in the improvement of energy status and thereby in protection of plants in the conditions of hypoxic stress.

## Functionality of Mitochondria Under Hypoxic Stress

The primary function of mitochondria is to generate ATP, thus these organelles are vital for plant survival. Since oxygen is essential for ATP, any change in its concentration can affect ATP levels; therefore it can affect all energy requirements for biochemical reactions in the cell. Since mitochondria take up oxygen for respiration, these organelles efficiently sense oxygen. Plant tissues, especially roots, experience hypoxia during flooding and waterlogging (Bailey-Serres and Voesenek, 2008). Plants experience hypoxia in roots, germinating and developing seeds and in any bulky tissues due to the restricted diffusion of oxygen into these tissues through internal cell layers (Tschiersch et al., 2011). During hypoxia, cytochrome oxidase (COX) will have a limited capacity to function using oxygen while another terminal oxidase, the alternative oxidase (AOX), practically does not function under hypoxia (Igamberdiev and Hill, 2009). This is due to the different *K*m values of these two terminal oxidases. For instance, the *K*m value of COX for oxygen is in the range from 0.1 to 0.15 μM whereas the *K*m value for AOX is in the order of 10 μM (reviewed in Igamberdiev and Hill, 2009). The lack of the terminal acceptor alters the mitochondrial functionality under hypoxia and near anoxia and results in impairment of the mitochondrial infrastructure (Vartapetian et al., 2003). The direct effect of oxygen deficiency on mitochondria is related to the lack of terminal electron acceptor in the electron transport chain and to the lack of ATP production. We will show below that plant mitochondria may have a sufficient metabolic plasticity to partially overcome these shortcomings, but their operation in such stress conditions is associated with the formation of reactive oxygen and nitrogen species.

## Plant Mitochondria Apply Various Strategies to Survive Under Hypoxia

Survival under hypoxia depends on energy production. Therefore it is very important for the cells to safeguard mitochondria during stress. Plant mitochondria adapt various strategies to retain their structure for prolonged periods of time. One of such strategies is to keep the production of ATP under low oxygen conditions. A preliminary clue about the requisition for ATP for structural integrity came from study by Coueé et al. (1992), where the exogenous supply of ATP was shown to result in the support of endogenous ATP production and protection of mitochondria. Later several studies revealed that the application of nitrate has a protective role for root mitochondria from maize and pea seedling (Müller et al., 1994; Vartapetian and Polyakova, 1999). These authors suggested that nitrate (NO_3_^-^) can act as a terminal electron acceptor that supports the operation of electron transport chain in the absence of oxygen. But so far there is no evidence that nitrate can act as a terminal electron acceptor. How nitrate can be transported to mitochondria is also not known. Plants take up nitrate by high and low-affinity transporters (encoded by the families of genes *NRT1* and *NRT2*) (Dechorgnat et al., 2011). After being taken up by roots, NO_3_^-^ is reduced first to NO_2_^-^ by the cytosolic nitrate reductase (cNR) where NAD(P)H is used as electron donor, and further the plastidal nitrite reductase reduces nitrite to ammonium (Simontacchi et al., 2015). But there are no reports that mitochondria contain any nitrate transporter. On the other hand, nitrate could indirectly support the functionality of seedling mitochondria under hypoxia via its reduction to nitrite, which can be imported to mitochondria by a similar transporter to that found in chloroplasts (Sugiura et al., 2007) or by the mitochondrial inner membrane anion channel (PIMAC) activated under low ATP conditions (Laus et al., 2008). In potato tuber mitochondria, 29 metabolite transporters have been identified (Salvato et al., 2014), while 58 members of the mitochondrial carrier protein family were described in *Arabidopsis* (Palmieri et al., 2011), and 50 members in rice (Taylor et al., 2010). Some of these transporters can potentially carry nitrite, however, further studies are needed to establish what particular carrier is used to import nitrite into mitochondria.

The mitochondria of some anoxia-tolerant plant species do not change their structure under anoxia. For instance the mitochondria of anoxia-tolerant plants *Echinochloa phyllopogon* and *E. crus-galli* retained their structure and metabolic activity during the prolonged exposure to anaerobic stress (Kennedy et al., 1987). In another study it was shown that the mitochondrial biogenesis did not alter in the anoxia-tolerant rice (Howell et al., 2007). All this suggests that plants can use a specific strategy to survive under hypoxia or anoxia by keeping their mitochondria in a functional state (Gupta and Igamberdiev, 2011; Igamberdiev et al., 2014; Shingaki-Wells et al., 2014).

## Mitochondria Can Reduce Nitrite to NO

Several lines of evidence suggest that mitochondria of different and maybe all species are capable of reducing nitrite to nitric oxide (NO). For instance, the mitochondria isolated from ciliate protists and Fusarium fungus, possess a capability to reduce nitrite to NO (Tielens et al., 2002). Kozlov et al. (1999) showed that rat liver mitochondria can produce NO using nitrite. The first evidence for this reaction in photosynthetic organisms came from the study of the green alga *Chlorella sorokiniana* where the addition of nitrite resulted in NO formation and could be blocked by the inhibitors of the mitochondrial electron transport chain (Tischner et al., 2004). Later it was shown that tobacco cell suspensions are able to reduce nitrite to NO and that the application of mitochondrial inhibitors suppressed this reaction (Planchet et al., 2005). Then Gupta et al. (2005) conducted a detailed study and found that nitrite reduction to NO takes place in root mitochondria from various species, such as pea, barley, *Arabidopsis* and tobacco, and determined the *K*m value for nitrite reduction to NO (175 μM). This allowed estimating nitrite concentration needed for NO production. Since under hypoxia nitrite reduction to ammonium is inhibited (Botrel et al., 1996), the accumulated nitrite can act as a substrate for NO formation. This reaction is highly sensitive to oxygen, which has a *K*i value of approximately 0.05% or 0.6 μM (Gupta and Igamberdiev, 2011). Gupta and Kaiser (2010) demonstrated that this process occurs in the membrane but not in the matrix of mitochondria. The complexes III and IV of the mitochondrial electron transport chain were shown to be the sites for NO production.

$${{NO}_{2}}^{-}+{2H}^{+}+e^{-}\to NO+H_{2}O$$

The complex III can produce NO via a leakage of electrons to nitrite in a similar way as superoxide is produced by one-electron reduction of oxygen at this site. The mechanisms of nitrite reduction to NO by COX are still under investigation and several models are available for the explanation of this mechanism (reviewed in Gupta and Igamberdiev, 2011). The availability of oxygen, nitrite and NO determines the redox state of the COX center that contains heme *a_3_* and copper B (Fe*_a_*_3_Cu_B_) that in turn depends on the redox state of cytochrome *c*. In the absence of oxygen, Fe^2+^ donates the electron for nitrite reduction to NO. But still the concrete details of this mechanism remain speculative.

Other sites of nitrite reduction to NO in mitochondria may include cytochrome *c* itself and other hemeproteins (Basu et al., 2008). The proteins other than hemeproteins may also be involved in NO formation. The involvement of the AOX, which is a di-iron carboxylate protein, in NO production was suggested on the basis of the effect of its inhibitor, salicylhydroxamic acid (SHAM) on NO evolution from mitochondria (Planchet et al., 2005; Gupta and Kaiser, 2010). Some proteins of a similar iron structure are found to be effective in NO metabolism (Kurtz, 2007). However, NO production in alfalfa (*Medicago truncatula*) nodules was fully insensitive to AOX inhibitor propylgallate (Horchani et al., 2011). The effect of AOX inhibitors reported in several studies may be explained by their action on other proteins, including peroxidases and other hemeproteins (Brouwer et al., 1986).

## Nitrite Reduction to NO Leads to ATP Generation via Phytoglobin-NO Cycle

Under anoxia mitochondria produce significant amounts of NO (Gupta et al., 2005; Planchet et al., 2005). One interesting puzzle is the physiological role of the mitochondrial NO production. Previously it was suggested that COX may play a role in membrane translocation of protons during nitrite reduction to NO (Castello et al., 2006). Isolated mitochondria of barley and rice were able oxidize the externally applied NADH and NADPH under anoxia when nitrite was applied, and this oxidation resulted in the detectable ATP formation (Stoimenova et al., 2007). The build-up of ATP during the oxidation of NADH and NADPH by anoxic mitochondria was sensitive to myxothiazol and KCN suggesting that the operation of the complexes III and IV is essential for this process. The anoxic production of ATP constituted only 3–5% of the aerobic ATP generation. However, the ATP produced during glycolytic fermentation together with the mitochondrial anaerobic nitrite-driven ATP production can make a major contribution for hypoxic survival.

**FIGURE 1:**
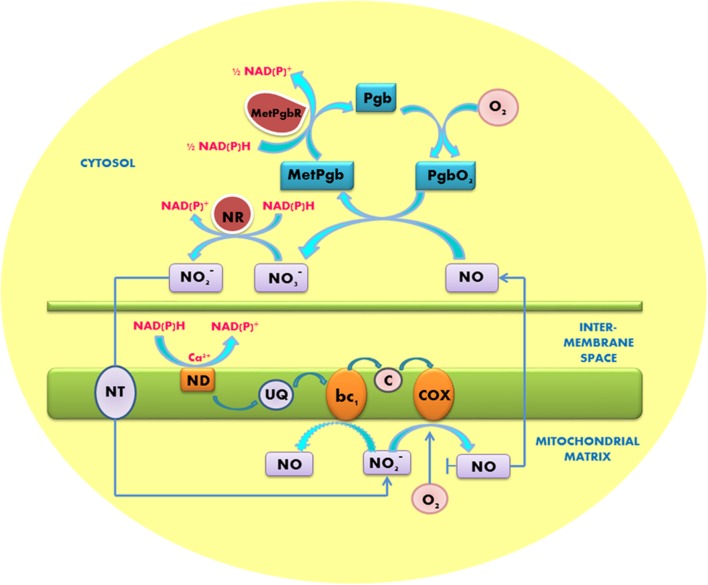
**Operation of the phytoglobin/NO cycle between the anoxic mitochondrion and cytosol.** Nitrite reduction and NO formation occur at COX and complex III (bc_1_). NO diffuses to the cytosol where it is converted to nitrate (NO_3_^-^) by the oxygenated non-symbiotic phytoglobin (PgbO_2_). Metphytoglobin (MetPgb) formed in this reaction is recycled by metphytoglobin reductase (MetPgbR) and the product nitrate is reduced by nitrate reductase (NR) to nitrite, which is transported to mitochondria by a putative nitrite transporter (NT). NAD(P)H is oxidized by the externally facing mitochondrial dehydrogenases (ND). Q, ubiquinone; c, cytochrome *c*. Modified from Gupta and Igamberdiev (2011).

The produced NO needs to be recycled very fast in order to avoid the nitrosative stress. Plants possess hypoxically induced hexacoordinated hemoglobins (called class 1 phytoglobins). They are the active scavengers of NO; thereby they can significantly reduce the amounts of NO to the levels that are much less toxic to plants. Class 1 phytoglobins possess the affinity to oxygen of two orders of magnitude higher than cytochrome *c* oxidase (*K*d ∼2 nM); therefore they can operate at the oxygen levels that cannot be utilized by mitochondria (Igamberdiev et al., 2011). Scavenging of NO involves the oxidation of phytoglobin ferrous ion, forming metphytoglobin and nitrate (Igamberdiev et al., 2006, 2011). Operation of the phytoglobin-NO cycle is essential for recycling nitrate and nitrite and for oxidation of excess of NADH and NADPH (Igamberdiev and Hill, 2004, 2009; Gupta and Igamberdiev, 2011; Igamberdiev et al., 2014) (**Figure 1**). This cycle can also contribute to NAD^+^ recycling for the continuous operation of glycolysis. Its operation is important not only under the hypoxic conditions (that occur during germination of seeds, in flooding stress or in compact meristemic tissues) but also in the course of pathogen infection, when significant amounts of NO are formed during the response mediated by salicylic and jasmonic acids (Mur et al., 2012).

## Is Nitrite-Driven Anaerobic ATP Synthesis Sufficient for Plants to Survive Under Hypoxia?

In order to survive under hypoxia and anoxia, plants need to sustain the machinery to carry out transcription and translation. It has been shown that the energy budgeting occurs under hypoxia to direct a specific amount of ATP for cellular functions (Edwards et al., 2012); at the same time cell recovery processes, such as cell division and elongation, decelerate in order to save energy (Takahashi et al., 2011). Several lines of evidence suggest that the active transcription of the mitochondrial genes takes place under hypoxia (Narsai and Whelan, 2013), while RNA translation appears to be downregulated. This was evidenced by 50% reduction in polysome content (Branco-Price et al., 2008) to reduce energy costs of unwanted translation during stress condition.

For instance, plants actively switch on the ethylene responsive transcription factors which are required for hypoxia sensing and survival (Bailey-Serres et al., 2012). In comparison to transcription, protein biosynthesis is much more energy demanding process. It has been shown that the translation of catabolic proteins takes place in anoxic coleoptiles of rice (Edwards et al., 2012). Plants invest energy in making catabolic proteins, such as the enzymes involved in glycolysis, under hypoxia/anoxia to get higher energy yield. Previously we have shown that mitochondria from roots produce NO within minutes of exposure to hypoxia suggesting that energy production initiation related to NO turnover starts within minutes. The anoxia-tolerant rice produced much higher levels of anoxic ATP in comparison to anoxia-intolerant barley suggesting essentiality of the nitrite-driven ATP production in the anoxic survival (Stoimenova et al., 2007). Since nitrate reductase (NR) is a part of the hemoglobin-NO cycle, its transcript is highly induced in anoxic rice coleoptiles (Lasanthi-Kudahettige et al., 2007). The increase of NR activity under oxygen deprivation (Planchet et al., 2005) suggests that the activation of transcription and translation of NR contributes to the excess of nitrite production and to NO generation, thus helping to improve the energy status of hypoxic plants to support the transcription and translation processes. The post-translational activation of NR under hypoxia is particularly important, which is achieved through dissociation of the 14-3-3 protein inhibitor and NR dephosphorylation (Allègre et al., 2004).

## ROS in Their Reaction with NO Produce Reactive Nitrogen Species (RNS)

Electrons enter the mitochondrial electron transport chain primarily via complex I, the alternative rotenone-insensitive dehydrogenases, and complex II (succinate dehydrogenase). These electrons are transferred to the ubiquinone pool which is a central reservoir for accumulation of electrons. From the ubiquinone pool electrons pass to complex III and complex IV, or to the AOX. The transfer through complexes III and IV leads to the generation of electrochemical proton gradient which is used by the ATP synthase to generate ATP (Rasmusson et al., 2008). When oxygen concentrations decrease below 10 μM, AOX does not function due its high *K*m to oxygen, and when oxygen concentrations fall further below 1 μM, the operation of COX is also ceased. This leads to the accumulation of electrons in the ubiquinone pool and at the other sites such as complex I and III. This results in the condition when the remaining oxygen molecules accept only one electron which leads to the production of superoxide anion (O_2_^-^). The elevated NADH/NAD^+^ ratio in the mitochondrial matrix under hypoxic conditions, becomes a condition leading to the increase in superoxide anion production even at low oxygen concentrations (Murphy, 2009). The reduced electron carrier proteins are able to react with O_2_ to form O_2_^-^, while NO produced under hypoxia can activate or repress these proteins to control O_2_^-^ production. NO is known to inhibit respiration by competitively binding to cytochrome c oxidase (Cooper, 2002). Thus, by increasing or decreasing the rate of O_2_ consumption by mitochondria NO may influence O_2_^-^ production *in vivo* by altering the local [O_2_] (Borisjuk et al., 2007). Recently it was shown that the overexpression of phytoglobins leads not only to the decrease in NO but also results in the increased respiration, lowering internal oxygen concentration, and subsequent production of ROS (Gupta et al., 2014). Paradoxically, the mitochondrial O_2_^-^ increases in response to low oxygen levels (Chandel et al., 1998; Guzy and Schumacker, 2006).

The produced superoxide is responsible for the generation of other ROS and of RNS. For instance, superoxide dismutase converts superoxide to hydrogen peroxide which can act as a signal. Its excessive amounts lead to cytotoxicity. NO does not directly react with H_2_O_2_ but there are reports that NO can reduce H_2_O_2_ formation (Małolepsza and Rózalska, 2005). This can be explained, in particular, by the activation through *S*-nitrosylation of ascorbate peroxidase, which is the key enzyme participating in H_2_O_2_ scavenging (Correa-Aragunde et al., 2013; Begara-Morales et al., 2014; Yang et al., 2015). The reaction between superoxide and NO occurs near the diffusion controlled rates and results in the formation of peroxynitrite (ONOO^-^), which is a toxic RNS form causing tyrosine nitration (Poyton et al., 2009). Peroxynitrite is formed in relatively low amounts under non-stress conditions, while under stress both NO and superoxide levels increase stimulating its formation. The biological reactions of NO and superoxide that limit their availability also constrain the amount of peroxynitrite formed. SOD competes effectively with NO for superoxide by reducing its level and decreasing the amount of peroxynitrite formed. The class 1 phytoglobin also decreases peroxynitrite concentration by reducing the availability of NO to react with superoxide. Peroxynitrite can be scavenged in plants via the pathway involving thioredoxin (Wulff et al., 2009), which results in its lower toxicity in plants as compared to animal tissues where its scavenging is likely limited by a side reaction of cytochrome *c* oxidase (Pearce et al., 2002). The excess of NO under hypoxia can react with peroxynitrite resulting in the formation of non-toxic nitrogen dioxide (NO_2_) and nitrite (NO_2_^-^). The produced NO_2_ can react with NO which then leads to the formation of dinitrogen trioxide (N_2_O_3_) which plays a role in nitrosative reactions (Espey et al., 2002).

In summary, plant mitochondria in the conditions of oxygen deficiency can reduce nitrite to NO, which can help in increasing their energy efficiency for supporting active transcription and translation processes in the hypoxic cells. NO participates in NAD^+^ recycling via the hemoglobin-NO cycle. In the reactions with ROS, NO forms peroxynitrite and other RNS such as N_2_O_3_ which play a role as signals during the nitrosative stress.

## Author Contributions

All authors listed, have made substantial, direct and intellectual contribution to the work, and approved it for publication.

## Conflict of Interest Statement

The authors declare that the research was conducted in the absence of any commercial or financial relationships that could be construed as a potential conflict of interest.
